# Development of functionalised polyelectrolyte capsules using filamentous *Escherichia coli* cells

**DOI:** 10.1186/1475-2859-11-163

**Published:** 2012-12-23

**Authors:** Franziska L Lederer, Tobias J Günther, Ulrike Weinert, Johannes Raff, Katrin Pollmann

**Affiliations:** 1 Helmholtz-Institute Freiberg for Resource Technology, Helmholtz-Zentrum Dresden-Rossendorf, 01314, Dresden, Germany; 2 Institute of Resource Ecology, Helmholtz-Zentrum Dresden-Rossendorf, 01314, , Dresden, Germany

**Keywords:** *Escherichia coli*, S-layer, Polyelectrolytes, Layer-by-layer (LbL), Palladium, SEM, TEM, Nanoparticle

## Abstract

**Background:**

*Escherichia coli* is one of the best studied microorganisms and finds multiple applications especially as tool in the heterologous production of interesting proteins of other organisms. The heterologous expression of special surface (S-) layer proteins caused the formation of extremely long *E. coli* cells which leave transparent tubes when they divide into single *E. coli* cells. Such natural structures are of high value as bio-templates for the development of bio-inorganic composites for many applications. In this study we used genetically modified filamentous *Escherichia coli* cells as template for the design of polyelectrolyte tubes that can be used as carrier for functional molecules or particles. Diversity of structures of biogenic materials has the potential to be used to construct inorganic or polymeric superior hybrid materials that reflect the form of the bio-template. Such bio-inspired materials are of great interest in diverse scientific fields like Biology, Chemistry and Material Science and can find application for the construction of functional materials or the bio-inspired synthesis of inorganic nanoparticles.

**Results:**

Genetically modified filamentous *E. coli* cells were fixed in 2% glutaraldehyde and coated with alternating six layers of the polyanion polyelectrolyte poly(sodium-4styrenesulfonate) (PSS) and polycation polyelectrolyte poly(allylamine-hydrochloride) (PAH). Afterwards we dissolved the *E. coli* cells with 1.2% sodium hypochlorite, thus obtaining hollow polyelectrolyte tubes of 0.7 μm in diameter and 5–50 μm in length. For functionalisation the polyelectrolyte tubes were coated with S-layer protein polymers followed by metallisation with Pd(0) particles. These assemblies were analysed with light microscopy, scanning electron microscopy, energy dispersive X-ray spectroscopy and transmission electron microscopy.

**Conclusion:**

The thus constructed new material offers possibilities for diverse applications like novel catalysts or metal nanowires for electrical devices. The novelty of this work is the use of filamentous *E. coli* templates and the use of S-layer proteins in a new material construct.

## Background

*Escherichia coli* are bacteria which naturally colonise the colon of mammalians. The typical cells of *E. coli* are rod-shaped with dimensions of 1.1-1.5 μm × 2.0-6.0 μm [1]. In molecular biology *E. coli* is generally used as a tool to produce proteins of interest of other organisms in a simple and high efficient way. In a previous study we described the formation of filamentous *E. coli* that are surrounded by tube-like structures consisting of outer membrane and surface (S-) layer proteins [2]. Although one of the best studied microorganisms only few reports describe the filament formation of *Escherichia coli*[3-6]. The formation of filamentous *E. coli* cells is in most cases a result of the inhibition of proteins that are naturally involved in bacterial cell division processes [7,8]. The previously described morphological changes were induced by the heterologous high level expression of the S-layer proteins of the uranium mining waste pile isolate *Lysinibacillus sphaericus* JG-A12. It was suggested that the expression of the S-layer protein SllB inhibits cell division and induces the secretion of these S-layer proteins to the surface of the *E. coli* cells. The stability of the filaments is a result of the S-layer proteins in the cell wall. The filaments that have a uniform thickness of 0.8-1 μm and can reach a length of several 100 μm have been discussed as promising bio-template e.g. for the production of catalytic active composites or metal microwires [2,9].

In previous studies cells of different organisms such as erythrocytes, bacteria and spores have been used as bio-template for the production of polyelectrolyte capsules [10-12]. Several studies describe the polyelectrolyte encapsulation and surface modification of living microbial and human cells. Protecting effects of these modifications against phagocytosis, increasing pH values or ultra violet radiation were analysed [13,14]. The stepwise polyelectrolyte adsorption to different materials such as cells or polymer particles is a useful way to create polymer multilayer films with defined chemical and physical properties. Decher and co-workers proposed this technique originally for the combination of linear polycations and polyanions [15,16]. The combination of multilayer systems with proteins was described later [17]. The starting material for this method is a solid substrate with a negatively charged planar surface. The formation of the first polyelectrolyte layer is started by addition and adsorption of cationic polyelectrolytes to the substrates. The adsorption is carried out at relatively high polyelectrolyte concentrations. A number of ionic groups remain exposed to the interface towards the solution that affects the effectively reserved surface charge. Substrate rinsing in pure water is followed by incubation of the substrate in an anionic polyelectrolyte solution. Multilayer assemblies are obtained by repeating these steps. Additionally, organic molecules and bio-components such as proteins, particles, bio-polymers and surfactants can be incorporated in these films, thus realising a multi-functionalisation of these layers [18].

In the present study we designed bio-based polyelectrolyte capsules by using filamentous *E. coli* as bio-template for the assembly of polyelectrolytes. The capsules were bio-functionalised by coating with bacterial S-layer proteins. S-layers are composed of two-dimensional, regularly arranged proteins or glycoproteins [19-21], which are the outer component of the cell wall of many bacteria and are a universal attribute of all archaea [22,23]. These proteins hold the ability to self-assemble into 2D arrays [24-26] and were found generally as mixture of monomer and polymer protein. Special characteristic of S-layers is the formation of regular arranged pores of identical size. S-layer proteins fulfil several functions like working as molecular sieve [27] and binding of toxic heavy metal ions [28-31]. The applications of S-layers is multifaceted and include the usage as ultrafiltration membranes [32], drug microcontainers [33], filter materials [34] or patterning structures in nanotechnology [35].

In the present study, bio-functionalised polyelectrolyte tubes were used as template for the bio-inspired synthesis of nanoparticular palladium as an example for hybrid material preparation. The outstanding effectiveness of the palladium nanoparticles as catalyst has received particular attention to this metal [36]. Wahl and others previously described the formation of biogenic palladium nanoparticles in pores of S-layer proteins at the surface of *Lysinibacillus sphaericus* JG-A12 [37]. Using the hydrogenation of itaconic acid Creamer and others demonstrated the superior catalytic activity of these materials [36]. Nanoparticles are very attractive for the development of new materials since their properties usually differ significantly from those of the bulk material. In particular, their physical behaviour can be drastically changed and the catalytic activity can be significantly enhanced due to the altered volume/surface ratio. The development of cluster-assembled materials with discrete, size-selected nanoparticles is of great interest to enable the fine-tuning of the properties of the nanoparticles. Especially the design of bio-nanohybrid materials by the combination of bio-molecules with nanoparticles is an emerging topic at the overlaps of biology, material sciences, and nanotechnology [38]. Previous studies describe the design of such bio-nanohybrid materials like the assembling of colloidal gold nanoparticles to the surface of growing fungi [39], the coating of polyelectrolyte encapsulated *E. coli* with gold and silver nanoparticles [40] or the functionalisation of bacterial cells using magnetic nanoparticles [41].

In our work we demonstrate the potential of the use of the S-layer induced filamentous cell structures for the construction of functional conductive metallic wires that potentially can be used for electronic devices or as new catalysts. The possibility to combine such inorganic structures with biological functions opens up new perspectives for multifunctional hybrid materials.

## Results

### Preparation of polyelectrolyte capsules

**Figure 1:**
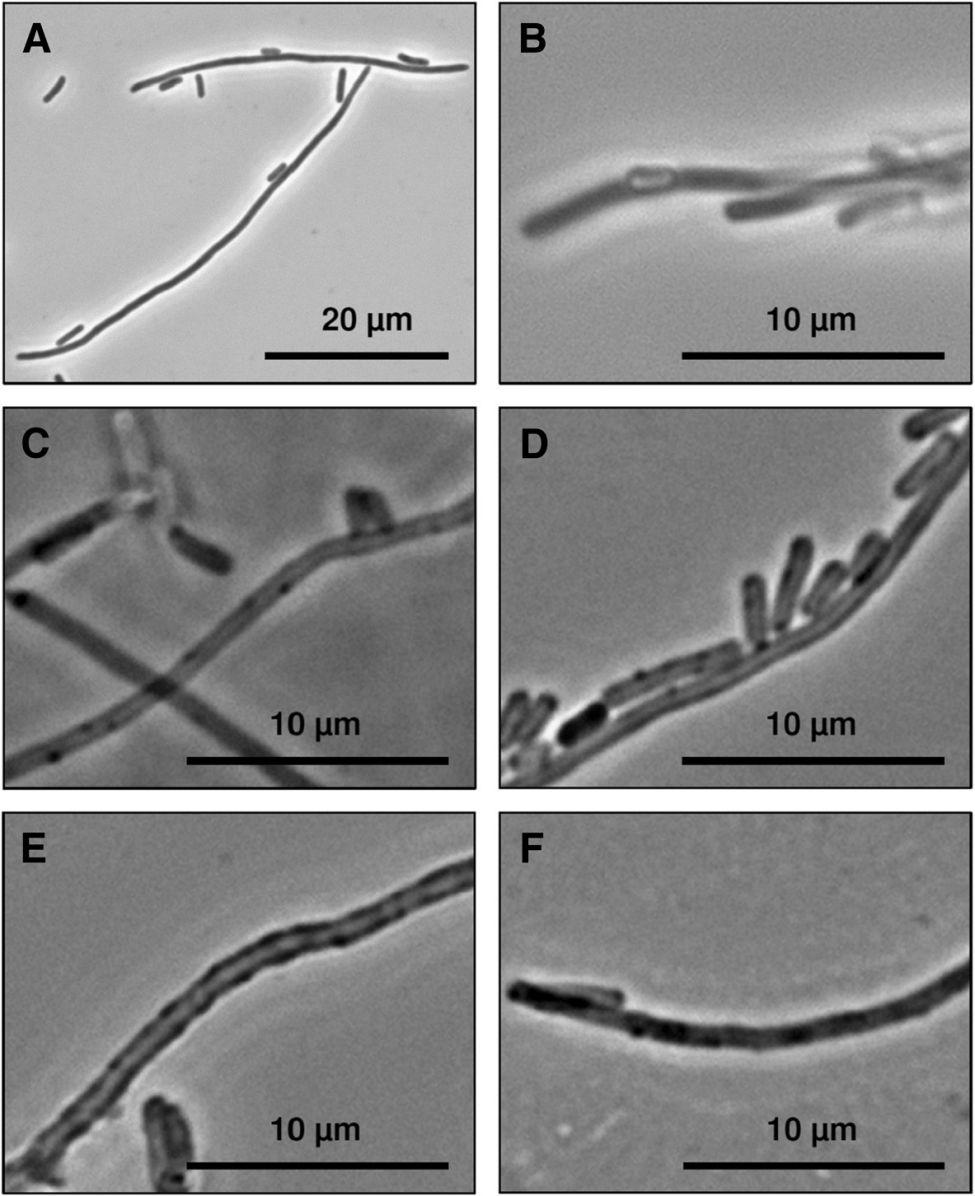
**Light microscope images of filamentous *E. coli *cells and polyelectrolyte capsules in phase contrast mode.** Image **A** presents filamentous *E. coli* cells in the exponential growth phase before polyelectrolyte coating. The polyelectrolyte coated *E. coli* filaments before NaOCl treatment are shown in image **B**. Image **C** presents polyelectrolyte tubes after the treatment with 1.2% NaOCl. The S-layer polymer protein coated polyelectrolyte capsules are shown in image **D**. Image **E** shows S-layer polymer protein coated polyelectrolyte tubes with synthesised palladium particles and image **F** presents polyelectrolyte capsules with synthesised palladium particles without S-layer proteins.

Coating of filamentous *E. coli* cells (Figure 1A) with the polyelectrolytes PSS and PAH followed by treatment with deproteinising NaOCl solution resulted in the formation of filamentous tubes (Figure 1C). Approximately one coated capsules per image section still contained bacteria (Figure 1D).

These tubes were in average 0.6-0.7 μm in diameter and 5–50 μm in length. Polyelectrolyte capsules showed marginal agglomeration and were stable for several days in deionised water at 4°C. The tubes were transparent and the presence of polyelectrolyte layers is indicated by higher contrast and more acute borders. For the development of the hollow polyelectrolyte capsules different parameters were tested. Especially the fixation of the *E. coli* filaments with glutaraldehyde in combination with the use of polycationic solution as first polyelectrolyte induced an irreversible agglomeration of the cells. In contrast, suspensions with well separated capsules were obtained when using a polyanionic solution as starting polyelectrolyte. For capsule preparation we tried different combinations of polyanions and polycations. The alternating coating of the cells with the polyanion PSS (poly(sodium-4styrenesulfonate) and the polycation PEI (Poly(ethylenimine)) caused severe cell agglomerations. Such agglomerations were avoided when using a combination of PSS as polyanion and PAH (poly(allylamine hydrochloride)) as polycation. In addition, agglomerations were prevented by thoroughly washing of the samples with 100 mM NaCl after each coating step and the pre-solution of the cells in 100 mM NaCl before each coating step.

### Coating of polyelectrolyte capsules with S-layer proteins

**Figure 2:**
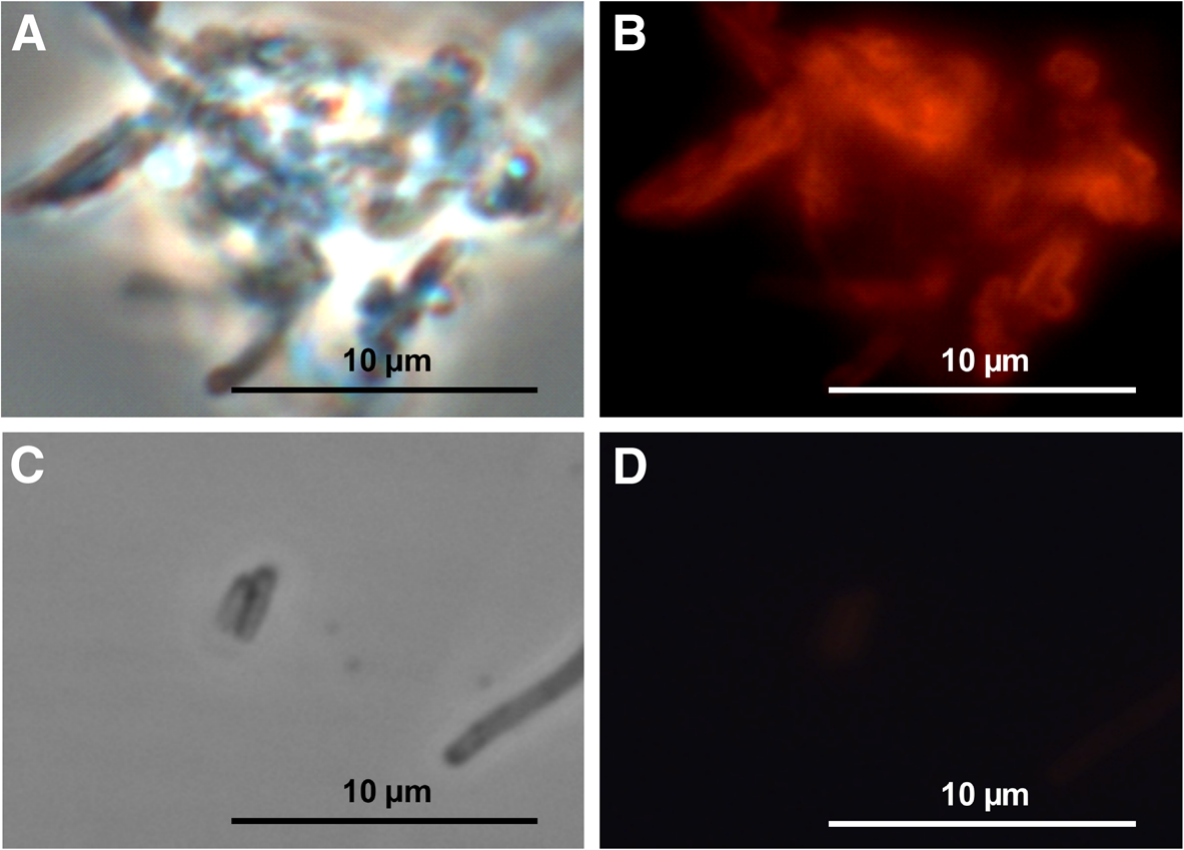
**Fluorescence microscopic images of S-layer coated polyelectrolyte capsules.** Images of filamentous polyelectrolyte capsules with HiLyte Fluor™ 488 amine linked S-layer polymer proteins in phase contrast mode (**A**), and excited by light in the 480–570 nm wavelength range using the filter U-MSWG (**B**). Polyelectrolyte capsules without S-layer proteins treated with HiLyte Fluor™ 488 amine in phase contrast mode are shown in image (**C**) and excited by light in the 480–570 nm wavelength range using the filter U-MSWG are shown in image (**D**).

The polyelectrolyte capsules were successfully coated with S-layer using a protein polymer solution as shown in Figure 2. Approximately 80 μg per millilitre S-layer polymer protein adsorb to the polyelectrolyte capsules and potentially the S-layer sheets form a monolayer at the surface. However, the degree of surface coating with S-layer polymer proteins is not known. To visualise the protein layers on the polyelectrolyte tube surface the proteins were coupled with the fluorescence dye HiLyte Fluor^™^ 488 and unbound fluorescence dye was removed prior to coating. Fluorescence microscopic images (Figure 2) present partial uniformly coated hollow capsule surfaces. S-layer polymer proteins labelled with fluorescence dye induce the reversible agglomeration of the coated capsules (Figure 2A, B). These analyses prove the formerly described high affinity of S-layer proteins to the polyelectrolyte tube surfaces [42]. In comparison, fluorescence dyes bound only sparse to polyelectrolyte capsules without protein coating as demonstrated by fluorescence microscopy (Figure 2C, D). Light microscopic analyses of S-layer polymer coated hollow polyelectrolyte capsules show nearly same proportions but few differences to uncoated polyelectrolyte tubes (Figure 1D). These S-layer coated polyelectrolyte capsules seem to exhibit more compact tube walls than those without proteins (Figure 1C, Figure 1D).

### Synthesis of Pd(0) particles

**Figure 3:**
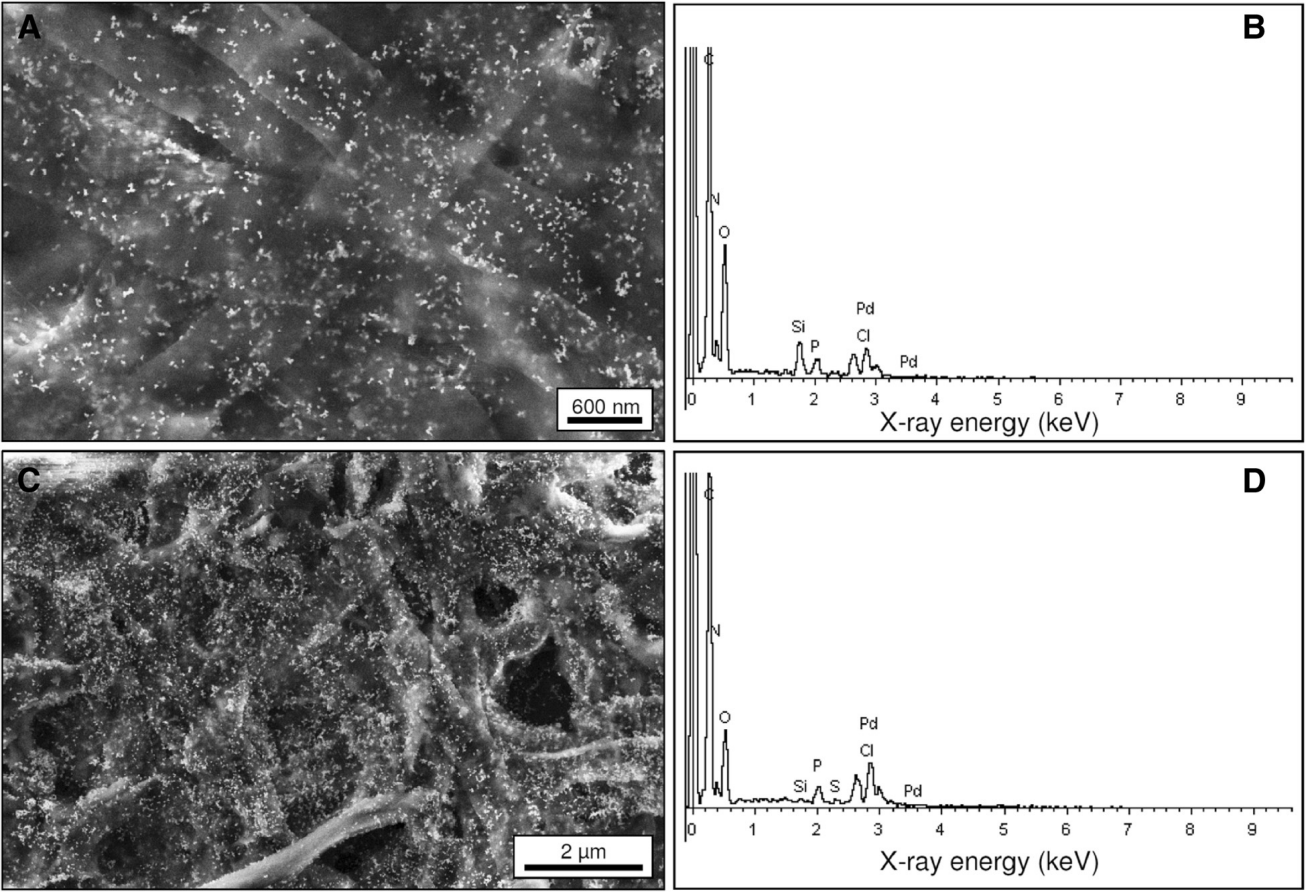
**SEM images of filamentous polyelectrolyte capsules.** Polyelectrolyte capsules without additional S-layer polymer protein with palladium particles are shown in (**A**) and with additional S-layer polymer protein with palladium particles are shown in (**C**). The images B and D present EDX analyses of parts of polyelectrolyte capsules with palladium particles without additional S-layer proteins (**B**) and with S-layer proteins (**D**).

**Figure 4:**
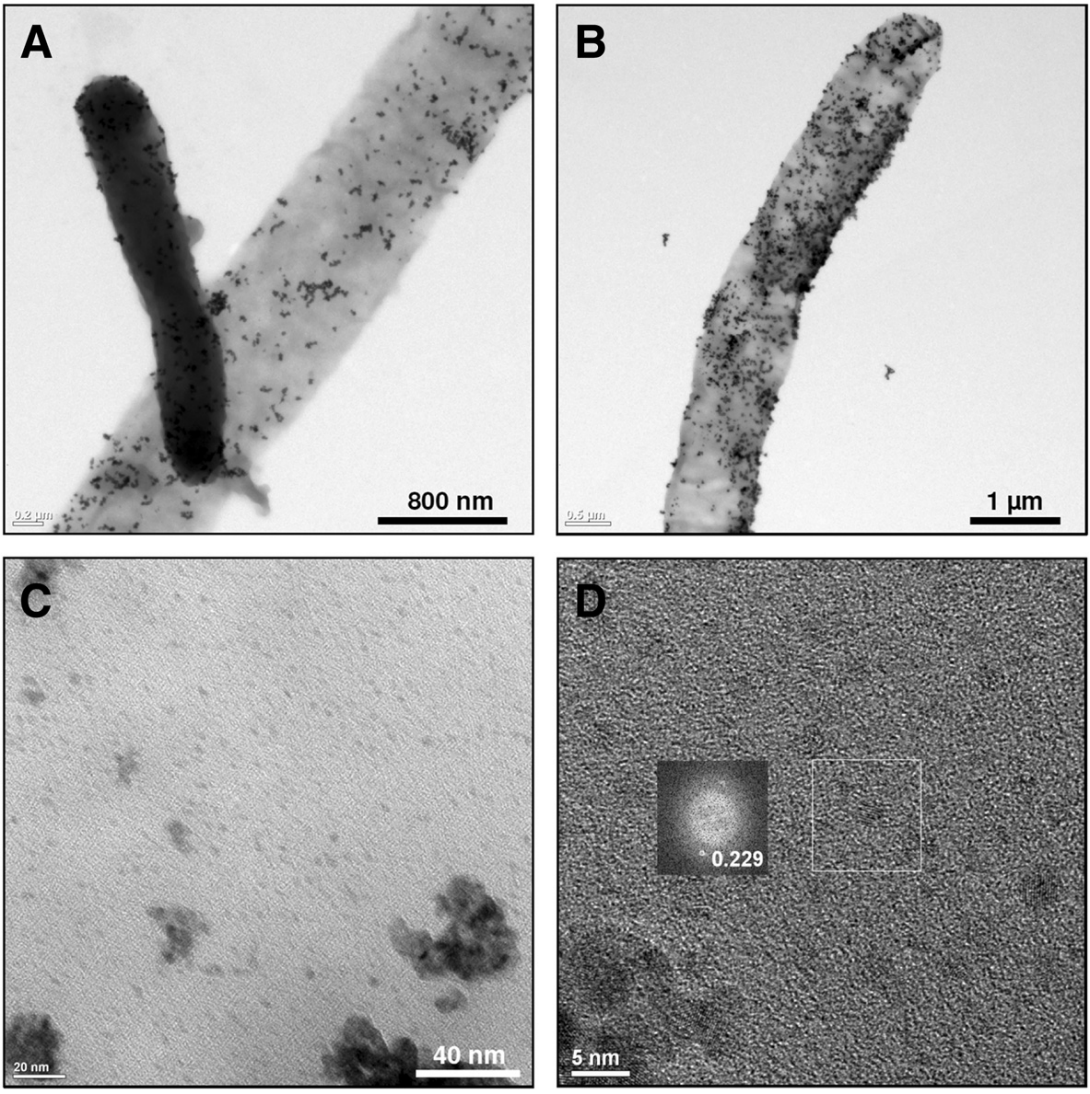
**TEM images of filamentous polyelectrolyte capsules.** Polyelectrolyte capsules without additional S-layer polymer protein with palladium particles are shown in **A** and with additional S-layer polymer protein with palladium particles are shown in **B**. The darker tube shows a filament that contains bacterial cells that were not removed during the procedure. TEM image **C** shows the surface of S-layer polymer protein coated polyelectrolyte tubes with crystalline palladium particles. In **D** a TEM micrograph of single Pd-particles and an insert of a Fourier transformation analysis of such a particle are shown. The latter indicates by the measured distance of the lattice planes the crystalline nature of these particles.

After incubation of S-layer coated polyelectrolyte capsules in Pd(II)-solution the tubes turned from colourless to brownish colour, indicating the binding of Pd(II)-complexes. After addition of the reducing agent, the brownish colour changed to black, indicating the formation of Pd(0). S-layer coated polyelectrolyte tubes with synthesised palladium particles are visible in the Figure 1E, Figure 3C and Figure 4B, however particles were identified distinct in the Figure 3C and Figure 4B. These tubes show uniform dark surfaces, pointing to the presence of Pd (0) (Figure 1E) and are in average 1–1.3 μm in diameter and 5–50 μm in length (Figure 4B). In order to get more information on particle formation, surface appearance but also the interior of the capsules the materials were investigated by SEM, EDX and TEM. These analyses (Figure 3C, Figure 4B) showed the presence of randomly distributed numerous dark (TEM) or white (SEM) particles with different sizes on the capsule surface. Further detailed images of the surface detected lots of smaller particles with diameters of 3–5 nm. Fourier transformation analyses of the TEM micrographs of formed particles demonstrated the existence of lattice planes in a distance of 0.225 nm, proving the existence of Pd(0) particles (Figure 4C-D). Larger particles of a size of 6–40 nm were identified by Fourier transformations as agglomerations of single palladium crystals with diameters of 5–6 nm (data not shown). The numbers and sizes of synthesised palladium particles are shown in Table 1. These analyses indicated a higher number of small palladium particles at the surface of polyelectrolyte capsules without S-layer proteins. However, those polyelectrolyte capsules with additional S-layer protein coating exhibit distinct higher numbers of large Pd particles.

**Table 1:** Palladium particle size analysis

**Samples**	**Particle size**	**Number of particles**
Polyelectrolyte capsules without S-layer proteins and with Pd	2-5 nm	0.16 particles per nm^2^
	> 5 nm	17.78 particles per μm^2^
Polyelectrolyte capsules with S-layer proteins and Pd	2-5 nm	0.063 particles per nm^2^
	> 5 nm	50 particles per μm^2^

The compositions of polyelectrolyte capsules without additional S-layer proteins with palladium particles (Figure 3B) and of polyelectrolyte capsules with additional S-layer protein with palladium particles (Figure 3D) were analysed by energy dispersive X-ray spectroscopy (EDX). Among other things like carbon, nitrogen and oxygen the analyses verified the presence of palladium. As reference, capsules without protein coating were used as template for the synthesis of Pd(0) particles. These materials are presented in Figure 1F, Figure 3A and Figure 4A. These tubes are 0.8-1.1 μm in diameter and 5–50 μm in length. Similar to the protein coated samples larger particles are visible at the surface of the uncoated capsules. However, in contrast to the protein samples, these particles are formed to a significantly less amount and showed a lower density.

## Discussion

Previous studies discovered the formation of unusual long *Escherichia coli* cell filaments induced by S-layer protein expression [2,9]. Such biological structures provide a promising matrix for technical applications such as the development of microcontainers or hollow metallic microwires. Especially gram-negative cells like *E. coli* are attractive for such applications. They possess a comparatively fragile cell wall that can be easily destroyed. *E. coli* can be easily cultivated giving a high yield of biomass and can be used for multifaceted applications. In the present study we used the cells for the synthesis of polyelectrolyte hollow capsules and investigated the possibility to use them as substrate for the functionalisation with proteins and metal nanoparticles.

The development of polyelectrolyte capsules was investigated by several groups using different kinds of templates postulating that those capsules are ideal candidates for applications in the areas of drug delivery, sensing and catalysis [38]. Sukhorukov and co-workers coated polystyrene and melamine formaldehyde latex particles with polyelectrolyte multilayers and dissolved the core [43], while Yu and others described the production of polymeric capsules with pre-loaded proteins based on mesoporous silica capsules which were finally removed [44]. The encapsulation of spores was described by Balkundi and co-workers aiming the development of environmental compatible materials for agriculture [12]. Franz and others investigated the encapsulation of microbes with different polyelectrolyte combinations and the following substrate uptake properties of enclosed bacteria [11]. These studies used the benefit of layer-by-layer technique which enables the variation of thickness, composition, and function of these assemblies by tuning the layer number, the species deposited, and the assembly conditions [38].

The present study describes the development of polyelectrolyte hollow tubes based on S-layer expressing *E. coli* cells which were fixed in glutaraldehyde and combined with the polyelectrolytes PSS (sodium poly(styrene sulfonate)) and PAH (poly(allylamine hydrochloride)) and a final NaOCl treatment. Other papers that used cells as template described the combination of negatively charged surfaces which were afterwards coated with the polycation followed by washing steps and a polyanion [15]. In contrast, the assembly of polyelectrolyte layers on *E. coli* filaments necessitated the starting with a polyanion to a probably negatively charged cell surface [45]. The combination of the glutaraldehyde fixed cells with polycationic solution induced an irreversible agglomeration of the cells. In comparison they stayed in suspension well separated when they were initially incubated with a polyanionic solution. Responsible for cell agglomerations which were observed after polycation incubation are potentially single positive groups at the mainly negative charged bacterial cell surface. Potentially, in the presence of polycations very high attractive forces operate between these cells which lead to agglomerations. However, negative polymers will saturate the few positive groups at the bacterial cell surface resulting in a very consistent charge distribution. So, the negative polymer works potentially as solubiliser.

Moya et al. described that treatment of polyelectrolyte encapsulated cells with NaOCl solution changed the chemical composition of the capsules dramatically. They observed the oxidation of the amino groups of polyallylamine to nitriles, nitroso-, nitro-, azo- and carbonyl groups and the disappearance of positive charges. Coevally the polymer chains were cross-linked with covalent bonds. Finally, the amount of PSS is strongly reduced to 10% of the original value. Moya et al. justified the stability of these capsules with the combination of cross-linking and hydrophobic interaction [45]. In our work, the use of the polyelectrolytes PSS and PAH in combination with sodium hypochlorite resulted evidently in the formation of uniformly coated stabile filamentous hollow capsules. However, round about 1% of the coated cells remain intact during NaOCl treatment. This observation leads to the assumption that these cells were not treated efficiently with NaOCl, perhaps because of their localisation in the lid of the reaction tube during incubation.

The surface coating of these tubes with surface layer polymer proteins aimed the synthesis of two dimensional crystal lattice which hold regular ordered nanopores with uniform bonding characteristics. Toca-Herrera and co-workers described the recrystallisation of S-layer proteins on polyelectrolyte surfaces and demonstrated by AFM that the combination of a final PAH layer with surface layer proteins hinder the recrystallisation of the proteins [42]. However, our light microscopic studies indicate that the binding of S-layer polymer proteins to polyelectrolyte capsules is enhanced with PAH as final polyelectrolyte capsule coating. It can be assumed that the constitution of PAH was influenced by sodium hypochloride treatment. Probably the uniform negative charges of the polyelectrolyte surface support the binding of S-layer polymer proteins via electrostatic attractive forces. The complete S-layer coating of the polyelectrolyte capsule surface is not assumed. S-layers were used to bio-functionalise the new-designed polyelectrolyte tubes.

In previous works self-assembling of bio-molecules to capsules or filaments has been reported several times and methods to functionalise these structures have been established. Mbindyo and co-workers reported the DNA-directed assembly of gold nanowires 0.2 μm in diameter and up to 6 μm in length [46], while the recognition capabilities of DNA, which induced the targeted attachment of functional wires were described by Braun and others [47]. Vauthey and co-workers described the molecular self-assembly of surfactant-like peptides to form nanotubes and nanovesicles [48]. The ability of protein coated peptide tubules to recognise and bind the protein complementary molecules in solution was investigated by Douberly and co-workers [49], while Yang and others analysed microtubules as templates for fabricating metallic nanowires [50]. Sugunan and others describe the formation of microwires of gold nanoparticle coated hyphea of *Aspergillus* strains while growing of initial spores in colloidal gold solution [39]. The assembly of nanoparticles on filamentous fungi generates microwires with extraordinary length. However, the diameter of the distinct shorter *E. coli* filament based polyelectrolyte capsules is smaller. The removal of the inner organic material of the *E. coli* filaments is much easier than the one of gold nanoparticle encapsulated filamentous fungi. The final synthesis of palladium nanoparticles in the pores of S-layer polymer proteins seems to produce distinct smaller nanoparticles than the glutamate stabilized gold nanoparticles. Kahraman and others studied the polyelectrolyte encapsulation of *E. coli* and *Staphylococcus cohnii* with additional gold and silver nanoparticles [40] while Zhang and co-workers analysed the functionalisation of bacterial cell walls with magnetic nanoparticles [41]. Fakhrullin and co-workers gave in their review a detailed overview over the studies which focus the functionalisation of living cells with polymers and nanoparticles [13].

The application of surface layer proteins as template for the synthesis of nanoparticles is a well established method [35,37,51]. S-layers are an interesting starting material for the synthesis of bio-inorganic composite materials that are promising for various applications, e.g. catalysts [36]. The proteins that are decorated with catalytic active nanoparticles can be fixed on carrier materials. The S-layer properties (amino acid composition, array symmetry and pore size) determine the nanoparticle properties like size and distribution. In previous work EXAFS and ATR-FT-IR analyses proved that carboxyl groups of the proteins are involved in the binding of the Pd(II) complexes [35,51]. In the present study we used S-layer coated polyelectrolyte filaments as carrier material for synthesis of Pd(0) particles. The immobilised S-layer proteins are able to bind Pd(II) complexes, thus enabling the synthesis of palladium particles by the addition of a reducing agent.

The newly designed bio-functionalised polyelectrolyte tubes that are described in this paper are unique due to its starting material. Specific regulations of template organism, temperature and amount of activator induce the formation of *Escherichia coli* filaments with defined diameter and cell wall stability. The template bacteria provide up to several 100 μm long structures with defined 0.8-1 μm in diameter which were encapsulated by layer-by-layer method with polyelectrolytes. After removing the bacterial core these polyelectrolyte hollow capsules can be bio-functionalised with S-layer polymer proteins which support the synthesis of metal nanoparticles in the protein pores. In conclusion, these filamentous polyelectrolyte tubes may provide an interesting matrix for the development of microcontainers and metal microwires with possibly novel physical and chemical properties. In combination with S-layer coupled palladium nanoparticles these materials could find application as novel catalysts or in the preparation of conductive metal microwires in electrical devices. Such developments are part of future work.

## Conclusion

In the present study we describe for the first time the use of filamentous *E. coli* as template for the assembly of polyelectrolytes. A method was developed that enables the synthesis of polyelectrolyte wires with a uniform diameter. These hollow fibres can be functionalised with proteins as well as with Pd(0) particles. These features make the filaments promising for future developments such as novel catalysts or metal nanowires for electrical devices.

## Methods

### Strains and culture conditions

*Escherichia coli* BL21(DE3), that express the S-layer protein SllB of the uranium mining waste pile isolate *Lysinibacillus sphaericus* JG-A12, were routinely grown in LB medium supplemented with kanamycin (35 μg ml^-1^) and 100 μM IPTG at room temperature for approximately 24 hours.

### Preparation of polyelectrolyte capsules

*Escherichia coli* cells were harvested in the stationary phase at OD_600_=2 and a pellet of at least 100 mg biomass was washed twice with 1 ml of 100 mM NaCl solution pH 7. The cells were fixed in the following step in 1 ml of 2% glutaraldehyde (Serva, Heidelberg, Germany) at room temperature for one hour as described elsewhere [45,52]. Afterwards the fixed cells were washed twice in 1 ml of 100 mM NaCl solution pH 7. The polyelectrolytes (PE) sodium poly(styrene sulfonate) (PSS) (Sigma, Aldrich, St. Louis, MO) of *M*_w_ ~70,000 Da and poly(allylamine hydrochloride) (PAH) (Sigma) of *M*_w_ ~56,000 Da were dissolved to a concentration of 1 mg ml^-1^ in 100 mM NaCl (Roth, Karlsruhe, Germany) solution pH 7. The final pH value of PSS solution was pH 6 and the pH value of PAH solution was pH 5. Six layers of freshly prepared PSS and PAH solutions were adsorbed onto the cells in the presence of 100 mM NaCl beginning with the polyanion. Each coating step lasted 10 minutes and was followed by four washing steps with 100 mM NaCl. After each step the cell pellet was concentrated by centrifugation at 12,000 *g* at room temperature for 3–5 min. To avoid cell agglomeration the cell pellet was resuspended in 150 μl of 100 mM NaCl before addition of polyelectrolyte solution. In the following deproteinisation step with 1.2% NaOCl (Sigma) [10] the cells were destroyed, while the hollow polyelectrolyte capsules remained. Capsules were washed four times in 100 mM NaCl to remove residual NaOCl.

### Linking of fluorescence dye to S-layer proteins

The fluorescence dye HiLyte Fluor™ 488 amine (MobiTec, Göttingen, Germany) was chosen for labelling of the S-layer proteins. For coupling reactions the S-layer proteins were dissolved in 50 mM MES-buffer (pH 5.6) and linked with the help of 200 μM cross-linker EDC (1-Ethyl-3-(3-dimethylaminopropyl)carbodiimid) (Sigma) to HiLyte Fluor™ 488 amine. The reaction took two hours. Afterwards uncoupled fluorescence dyes were removed by centrifugation and fluorescence labelled S-layer protein polymers were washed with buffer.

### Coating of polyelectrolyte capsules with surface layer proteins

The natural S-layer proteins of *Lysinibacillus sphaericus* JG-A12 were purified as described elsewhere [34]. Briefly, the S-layer protein expressing cells were grown in NB medium at 30°C, harvested by centrifugation and washed. Bacterial flagella were removed by treating the cells with the rotating-blade blender IKA T8 (IKA Labortechnik, Staufen, Germany) and following centrifugation steps. The cells were disintegrated by using the high-shear fluid processor at a pressure of 960 bar (M-110S Microfluidizer processor, Microfluidics, Newton, MA, USA). The cell fragments were washed, treated with Triton X-100 and washed again. Peptidoglycan was lysed by treatment with lysozyme. The S-layer containing fraction was washed several times, mixed with guanidine hydrochloride and non- protein compounds were removed by centrifugation. In order to remove guanidine hydrochloride the S-layer containing supernatant was dialysed several times against 1.5 mM Tris and 10 mM CaCl_2_, pH 8. Recrystallised S-layer proteins were collected by centrifugation and stored at 4°C for later applications. The isolated S-layer proteins are of high purity and were found in balance as monomer and polymer proteins (U. Weinert, pers. communication).

The polyelectrolyte tubes were washed and resuspended in 1 ml of 10 mM CaCl_2_ solution. Subsequently, 200 μg ml^-1^ of native or fluorescence labelled S-layer polymers were added to the polyelectrolyte capsule solution and bound to the surface of the polyelectrolyte tubes. The solution was stirred at room temperature for 20–24 hours. Afterwards the solution was concentrated by centrifugation at 12,000 *g* at room temperature for 3–5 min and washed twice with distilled water. The supernatants were removed.

### Synthesis of Pd (0) particles

Pd(0) particles were synthesised as described elsewhere [35]. Briefly, the S-layer polyelectrolyte tubes were concentrated by centrifugation. The Pd-solution was prepared 24 hours before usage. For this 2 mM Na_2_PdCl_4_ (Sigma) were dissolved in water and incubated overnight in the dark. The coating was started by addition of 10 ml Na_2_PdCl_4_ solution to the polyelectrolyte capsules. After 4 hours of incubation at room temperature under shaking in the darkness the tubes were washed twice in deionised water. Afterwards the bound Pd(II) was reduced by the addition of 30 μl of 100 mM dimethylamine-borane (Merck, Darmstadt, Germany) [35]. The directly observed sample colour change indicated the successful reaction. The sample was centrifuged and the pellet was washed twice and finally stored in deionised water.

### Characterisation of polyelectrolyte capsules and Pd (0) particles

Surface texture, height and uniformity of the polyelectrolyte coated capsules and the Pd(0) particle structures were analysed by light microscopy, scanning electron microscopy (SEM), transmission electron microscopy (TEM) and energy dispersive X-ray spectroscopy (EDX). Light microscopic images of cells and polyelectrolyte capsules were taken with the Olympus BX61 microscope (Olympus, Hamburg, Deutschland) in phase contrast mode. Fluorescence microscope images were taken with the filters U-MSWG (480–570 nm) and U-MNIBA (470–525 nm). Scanning electron microscopy (SEM) images of polyelectrolyte capsules and Pd(0) particles were obtained using the crossbeam workstation NVision 40 (Carl Zeiss SMT, Germany) at 5 keV. The morphology and chemical composition of the polyelectrolyte capsules and the Pd(0) particles was evaluated using a Titan 80–300 transmission electron microscope (FEI, Eindhoven, The Netherlands) at 300 keV. Energy dispersive X-ray spectroscopy (EDX) analyses were obtained after activation scanning electron microscopy (SEM) with the EDX system Quantax 400 (Bruker AXS, Karlsruhe, Germany) with the Si-drift detector XFlash 123 eV.

Samples for scanning electron microscopy investigations were applied to RCA purified Si wafers, each [53]. Samples were dried for about 24 hours at room temperature and analysed later with the scanning electron microscope. For transmission electron microscopy the samples were dried for about 24 hours at room temperature on carbon-coated copper grids.

## Abbreviations

S-layer: Surface layer; PAH: Poly(allylamine hydrochloride); PSS: Poly(sodium 4-styrenesulfonate); SEM: Scanning electron microscopy; TEM: Transmission electron microscopy; EDX: Energy dispersive X-ray spectroscopy.

## Competing interests

The authors declare that they have no competing interests.

## Authors’ contributions

FLL performed all the experimental work and wrote the manuscript. TJG advised to perform the surface coating with polyelectrolytes. UW provided the fluorescence dye labelled S-layer proteins and advised to perform the coating. JR conceived of the study. KP conceived of the study and was involved in drafting the manuscript. All authors were involved in the ongoing scientific discussion as well as all read and approved the final manuscript.
